# Cardiac Rehabilitation Based on the Walking Test and Telerehabilitation Improved Cardiorespiratory Fitness in People Diagnosed with Coronary Heart Disease during the COVID-19 Pandemic

**DOI:** 10.3390/ijerph18052241

**Published:** 2021-02-24

**Authors:** Ladislav Batalik, Vladimir Konecny, Filip Dosbaba, Daniela Vlazna, Kristian Brat

**Affiliations:** 1 Department of Rehabilitation, University Hospital Brno, 62500 Brno, Czech Republic; dosbaba.filip@fnbrno.cz (F.D.); vlazna.daniela@fnbrno.cz (D.V.); 2 Department of Public Health, Faculty of Medicine, Masaryk University, 62500 Brno, Czech Republic; 3 Non-Government Health Facility, Cardiovascular Rehabilitation, 62500 Brno, Czech Republic; konecny.edu@email.cz; 4 Faculty of Medicine, Masaryk University, 62500 Brno, Czech Republic; brat.kristian@fnbrno.cz; 5 Department of Respiratory Diseases, University Hospital Brno, 62500 Brno, Czech Republic

**Keywords:** cardiac telerehabilitation, cardiac rehabilitation, COVID-19, physical exercise, coronary heart disease, cardiorespiratory fitness, heart rate monitor

## Abstract

This study investigated an alternative home-based cardiac telerehabilitation model in consideration of the recommendations for the COVID-19 quarantine of people diagnosed with coronary heart disease (CHD). We hypothesized that using a 200 m fast walking test (200 mFWT) and telerehabilitation would create an effective alternative cardiac rehabilitation (CR) intervention that could improve cardiorespiratory fitness. Participants (*n* = 19, mean age 60.4 ± 9.6) of the 8-week intervention performed regular physical exercise at the target heart rate zone determined by calculations based on the 200 mFWT results. In our study, the participants were supervised using telerehabilitation. A total of 84% of participants completed the 8-week intervention. No adverse events were reported during telerehabilitation. The study participants noted a significant improvement (*p* < 0.001) in cardiorespiratory fitness expressed by an 8% reduction in the walking test time (Δ 8.8 ± 5.9 s). Home-based telerehabilitation based on 200 mFWT effectively increased the cardiorespiratory fitness in people with CHD with a low to moderate cardiovascular risk. This was a novel approach in CR during the COVID-19 pandemic. As research in this area is justified, this paper may serve as an alternative method of providing healthcare during the COVID-19 pandemic and as a basis for further upcoming randomized controlled trials.

## 1. Introduction

Coronary heart disease (CHD) is the cause of a large proportion of all cardiovascular deaths worldwide [1]. Recommendations for secondary prevention emphasize various approaches to cardiovascular risk management [2]. Physical exercise has been consistently identified as an integral part of cardiac rehabilitation (CR) [3]. For people diagnosed with CHD, training can improve their cardiorespiratory fitness, quality of life and reduce the mortality and number of rehospitalizations [4]. Despite the recognized benefits of CR, many barriers limit the use of outpatient CR exercise programs [5].

Telerehabilitation is an alternative approach that could alleviate some of these barriers. Telerehabilitation includes providing distance rehabilitation services via information and communication technologies (ICT), such as telephone, Internet, and video conferencing [6]. This model has been successfully studied in people with various cardiopulmonary diseases and is now being promoted as a possible part of standard healthcare [7,8,9].

Practically the entire world was struck in 2020 by the SARS-CoV-2 pandemic, which caused the COVID-19 illness [10]. In the acute phase of the COVID-19 pandemic from March to May 2020, a significant part of elective and outpatient healthcare was reduced or postponed for safety reasons. In particular, CR training programs and stress tests were scaled down due to concerns regarding the disease’s spread due to the potential transfer of aerosol particles during exercise [11,12]. Therefore, in many centers or specialized CR clinics, the programs were partially interrupted. During the summer, the CR centers were reopened, but with the onset of autumn, restrictions were reintroduced, calling for recommendations for a remote approach offered by telerehabilitation [13].

In particular, due to the quarantine and isolation during the COVID-19 pandemic, public health measures were associated with anxiety and unhealthy lifestyles, including physical activity limitations and an unhealthy diet [14]. Therefore, there is an urgent need to increase the level and reorganization of CR services and secondary prevention [15].

This study aimed to investigate an alternative model of home-based cardiac telerehabilitation (HBCT) considering the recommendations for COVID-19 quarantine in people diagnosed with CHD. To our knowledge, this is one of the first studies to examine the effect of alternative CR during the COVID-19 pandemic. We assumed that with the help of the walking exercise test and telerehabilitation, we would be able to create an effective alternative CR intervention to improve cardiorespiratory fitness.

## 2. Materials and Methods

From October 2020 to January 2021, we performed an 8-week mono-centric study of HBCT in people diagnosed with CHD at the University Hospital Brno, Czech Republic. Due to government regulations of the Czech Republic, which included state of emergency and restrictions of group exercise programs, the HBCT study was designed without a control group of participants [16]. The study corresponded to the Helsinki Declaration of Ethical Principles for Medical Research, in which human subjects participated, and was approved by the Ethics Committee of the University Hospital Brno, Czech Republic, under registration number 02-061020/EK. Participants signed an informed consent form before entering the study.

**Figure 1:**
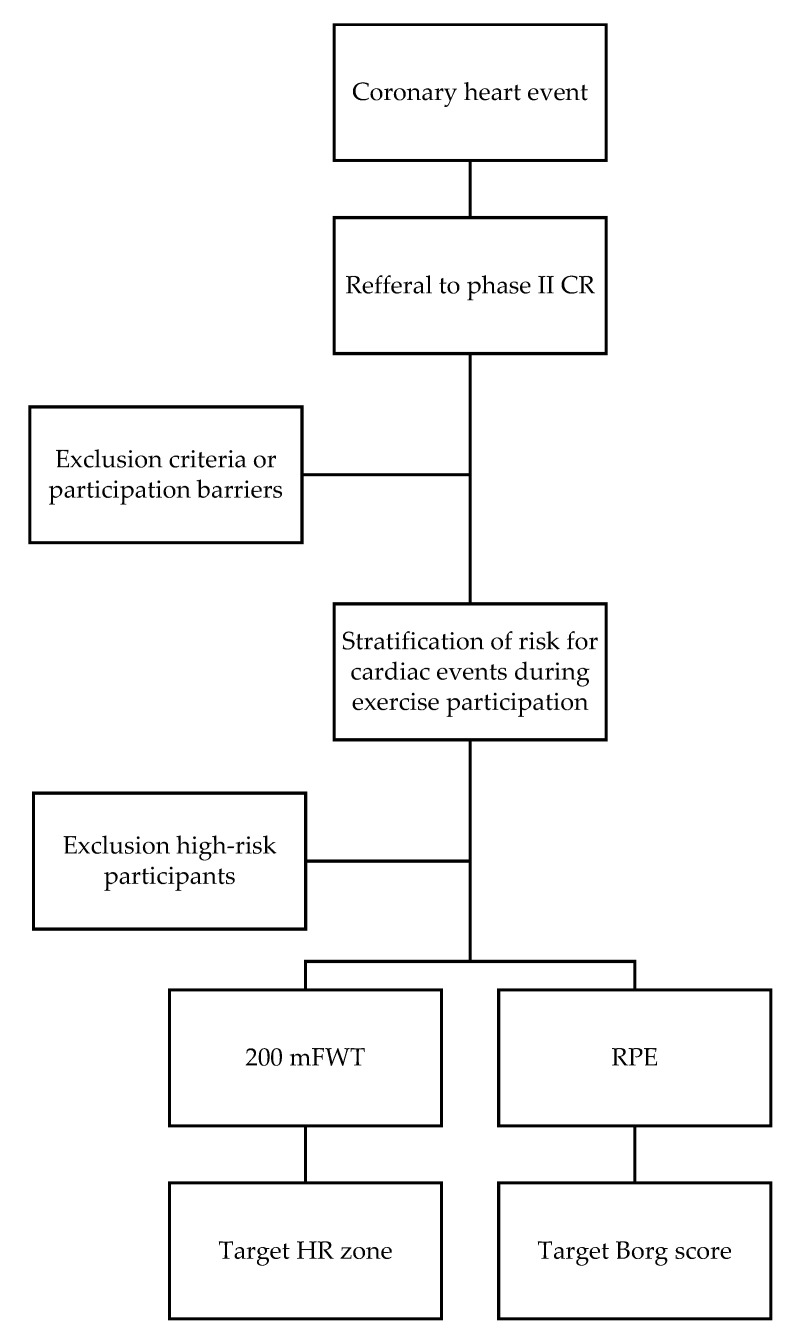
Decision flow chart.

The research participants (*n* = 19, women 5 of 19) were referred by medical staff to Phase II CR. The participants were of European descent, over 18 years of age, diagnosed with CHD (myocardial infarction in the last two months), and with low to moderate cardiovascular risk according to the American Association of Cardiovascular and Pulmonary Rehabilitation (AACPR) [17] (Figure 1). All participants of the HBCT were post-cardiovascular revascularization and were introduced to the recommended pharmacological treatment.

The clinical conditions allowed the participants to undergo a pedestrian stress test. The participants were able to understand and write in the Czech language. Another entry criterion was ICT ownership (personal computer, telephone or mobile connection, and Internet access) and literacy in its operation. The recommended participants of the HBCT study group were new participants entering phase II CR or current participants in an outpatient phase II program who agreed to the study and had completed less than two weeks of supervised exercises. Participants meeting the above conditions signed an agreement to participate and were included in the HBCT study.

The intervention was based on the principles of phase II CR and consisted of regular physical exercise and teleconsultations in the home environment of the participants. The 8-week HBCT intervention was aimed primarily at increasing cardiorespiratory fitness. The anamnesis (cardiovascular disease, treatment, risk factors for CHD, and anthropometric indicators) was taken from the participants in the form of a questionnaire. The HBCT study was initiated by a walking exercise test, education on the secondary prevention of CHD, and adherence to physical exercise (time, intensity, and rate of Perceived exertion—RPE). The participants were lent a Polar M430 heart rate (HR) monitor and an H10 chest sensor (Kempele, Finland) compatible with the web platform. Gilgen-Ammann et al. (2019) demonstrated the validity of the Polar H10 HR sensor in a study, where excellent HR variation intervals were detected during low-to high-intensity exercise activities [18].

All participants were introduced to the target Borg score for the current estimate of exercise intensity [19]. The participants were informed about the HR monitoring device and the possibility of sharing training data, for which a participant profile was created in the PolarFlow web platform. Access to the platform was granted only to the participant and the authorized physiotherapist using a shared password. The physiotherapist continuously checked the data and then collected it on an external secure hard drive. The benefit of using the web platform was the possibility to use the training diary, through which it was possible to view the data history of the training activity or compare training values.

Participants of the 8-week HBCT intervention were instructed to perform regular physical exercise 3–5 times per week, for a minimum of 30 min at the target HR zone determined by calculation based on the 200 m fast walking test (200 mFWT) result.

Target HR = 65 − 80% of calculated HRmax(1)

calculated HRmax = 130 − 0.6 × age + 0.3 × HR200mFWT(2)

The calculation was adapted according to the study of Cassilas et al. [20]. The participants were instructed that the intensity of the load corresponded to the RPE rating between “somewhat hard” and “hard” (12–14 on the target Borg score) [21]. The physiotherapist acted as a remote coach, who checked on the participants and provided them with telephone feedback once every two weeks.

Telephone feedback was arranged for a specific day and time. The physiotherapist contacted the monitored participant, with whom they consulted based on telemonitoring the completed training activity in terms of the intensity duration and answered questions related to the training, and positively motivated the participants for the next period. If necessary, the participants could also contact the physiotherapist in the CR center during the intervention. When detecting noncompliance with the prescribed training, the participants were contacted and advised to optimize their performance.

The participants were instructed to report any cardiovascular complications, cardiac events, and hospitalizations. After completing the 8-week HBCT intervention, all participants were tested in a walking stress test, and the training adherence was evaluated for each participant. Participants were excluded if they were unable to complete the HBCT intervention within 8 weeks or were unable to attend scheduled sessions of physical exercise.

### 2.1. Method of Data Collection

Study data were collected at the beginning of the intervention (T1) and after 8 weeks (T2). The primary measure of outcome was the change in cardiorespiratory fitness expressed as a change in the duration of the 200 m fast walking test (200 mFWT). Secondary results were measurements of the maximum achieved HR, restitution HR, and RPE of 200mFWT. The training adherence was recorded for each participant throughout the study. At the end of the intervention, the adherence was evaluated as the number of recorded training sessions in the web application using an HR monitor/sensor. The data on average HR as a percentage, average training session time in minutes, average number of sessions, and average RPE reported during training were used.

### 2.2. Two Hundred-Meter Fast-Walk Test

200 mFWT is a fixed distance walking test. A 50 m-long distance was laid out in the hospital. The corridor was ventilated, and a 2 m spacing between the assessor and the assessee was observed for preventive reasons due to the COVID-19 pandemic. Respiratory protective equipment was available only to the evaluator. The test aimed for the participant to cover a distance of 200 m as quickly as possible without running. (i.e., twice to cover a 50 m distance) Rest support was available in the middle of the track. Slowing down and stopping to rest was allowed. The time required to perform the test was measured in seconds at the end of the 200 m.

During the test, the participants’ HR was monitored using a Polar M430 and H10 monitor (Kempele, Finland). In the middle of each phase of the test, encouragement was provided for participants to practice until exhaustion. Participants were also asked to rate their RPE on a Borg scale (6–20) at the end of each test [21], and any clinical signs such as chest pain, dizziness, or musculoskeletal pain were noted. A six-minute recovery followed the 200 mFWT. The maximum HR was defined as the highest HR reached at the end of the 200mFWT. The restitution HR was recorded after a 6-min recovery period.

The 200 mFWT test corresponded to the intensity or submaximal effort. We found that feasible, well-tolerated, and reliable functional testing of stable individuals with CHD was feasible. The walking test is a simple model for evaluating aerobic capacity [22]. 200 mFWT was also investigated as an alternative to determine the target HR for cardiovascular training [20].

### 2.3. Rate of Perceived Exertion (RPE)

RPE is a method of evaluating and measuring the degree of intensity of physical exertion. Perceived physical exertion is based on physical sensations during exercise (increased HR, respiratory rate, and muscle fatigue). This is a subjectively evaluated parameter of exertion based on a scale from 6 to 20 and can provide an alternative way of regulating physical training intensity [21]. Under load, it is possible to evaluate the perceived effort ranging from “6” (“no effort at all”) to “20” (“maximum effort”). Based on subjective feelings during exercise, it is possible to help the RPE adjust the intensity by accelerating or slowing down the activity.

Experts from AACPR prescribing the home-based CR programs recommend the perceived effort during physical training should be between 12 and 14 on the Borg scale (i.e., the medium-level of intensity) [23]. Training at this range is as effective as conventional interventions based on the gold standard (determined by the percentage of HR reserve obtained from the cardiopulmonary exercise test) in people with CHD [24].

### 2.4. Statistical Method

Statistical analysis was performed using Statistica 12 statistical software (TIBCO Software Inc., Palo Alto, CA, USA). Values were expressed as the median value (standard deviation). The Wilcoxon test was used to compare 200 mFWT parameters in the T1 and T2 timelines. Statistically significant differences were considered at the recorded level of *p* less than 0.05.

## 3. Results

A total of 19 participants from the University Hospital Brno Czech Republic participated in the HBCT study. Six (32%) participants who had not completed more than two weeks of exercise were included from the outpatient phase of CR. Other participants were enrolled from the outpatient phase II CR program. The main characteristics of the participants included in the HBCT study are listed in Table 1. All participants were diagnosed with CHD, underwent cardiovascular revascularization, and received pharmacological medication of beta-blockers. Most participants were men with the typical cardiovascular risk factors.

The 8-week intervention was completed by *n* = 16 (84%) participants. The reasons for not completing the study (*n* = 3) were as follows: in one case, it was rehospitalization with percutaneous coronary intervention, another participant became ill with COVID-19 and remained in isolation and was subsequently not advised to continue, and in the latter case the participant refused to continue at their request.

The data in Table 2 refer to sixteen participants who completed the 200 mFWT at baseline and after 8-weeks of cardiac rehabilitation. The Wilcoxon signed-ranks test showed that participants statistically significantly improved their cardiorespiratory fitness by reducing the 200 mFWT time by 8% (Δ by 8.8 ± 5.9 s). The above increase in performance was achieved without a significant change in the HRmax at the end of the walking test or the mean RPE score on the Borg scale.

The results of the study participants’ adherence recorded an average of 3.4 ± 1.1 completed training sessions (ranging between 1.4 and 5.0). The average aerobic training duration was 40.1 ± 12.6 min (range 30 to 75 min). The average intensity of physical exercise was observed at the level of 95.1% ± 7.6% of HR achieved at the end of 200 mFWT, and the subjectively evaluated perceived RPE effort at the level of 13 ± 0.9. Of the total number of planned telephone consultations, 89% of calls were made. There were no physical exercise-related adverse events throughout the telerehabilitation intervention.

## 4. Discussion

In the current situation, where the main interest is to maintain public health during the COVID-19 pandemic, there were no clear recommendations on how to proceed in the rehabilitation of people diagnosed with CHD. The use of effective CR strategies based on the gold standard is problematic in current clinical practice, where CR is reduced or limited [15,25]. Innovative social connectivity methods during home-based CR are crucial to keeping participants motivated until the vaccine for SARS-CoV-2 is widely available. Although telemedicine is mostly unused in the management of cardiovascular diseases, the COVID-19 pandemic has reinstated interest in the use of innovative strategies in the provision of healthcare [26,27]. This means that HBCT can bridge the gap and be an effective alternative approach [28].

In our study, the participants were monitored remotely, with telerehabilitation, and, specifically, 8-weeks of exercises were monitored with a web application. The feasibility and efficacy of similar telerehabilitation approaches have been reported in people with CHD, and these approaches are strongly recommended as possible alternatives [13,29]. Therefore, our work is complementary because it combines the recognized alternatives of the walking test and remote telemonitoring in CR.

The results of the 8-week effect of HBCT intervention created the expected significant improvement in cardiorespiratory fitness by almost 8%, which corresponds to studies where the usual effect of exercise was around 7–15% [30,31,32]. It is necessary to mention that cardiorespiratory fitness is one of the independent factors predicting cardiovascular mortality, morbidity, and rehospitalization [33]. The results of the walking test’s secondary parameters for HR max and RPE did not show a significant change. This work had one chronotropic response in CHD participants treated with beta-blockers at higher walking speeds, which was noted at the beginning of the intervention. Similar results were demonstrated by Gremeaux et al. in a study that compared the walking test and the cardiopulmonary test in an outpatient CR model [34].

During the discussion of the compliance results, CR’s proposed forms and modalities in people with cardiovascular disease contributed to good compliance and subsequently led to improved functional capacity [35]). In our study, the overall dropout rate, followed by exclusion from the study that we recorded, was comparable to previous studies [8]. The participants understood the need to adhere to individual activities and cooperate with the telemonitoring center.

The recorded findings of training adherence are consistent with the study by Kraal et al., who used a similar monitoring strategy during telerehabilitation [36]. To summarize, our findings indicate that participants could self-adhere to the established home-based physical exercise when given adequate motivational guidance from a physiotherapist and an appropriate form of feedback. The research demonstrated that adherence to physical exercise was higher with the possibility of the participants determining their training location [37].

The safety of the participants is an essential issue of telerehabilitation and must be addressed. The studies published so far have shown that telerehabilitation exercise in people with CHD is safe [23]. Unified reports on HBCT in high-risk participants confirm that the remotely monitored persons’ safety, considering all indications and contraindications of such training, will be strictly observed, and interactive cooperation between the participant and the telemonitoring center will be ensured [38]. Our study did not record any adverse events during physical exercise, indicating that the telerehabilitation method was safe and feasible.

Our study attempted to create an effective procedure for offering optimal alternative interventions in the Czech Republic in a situation where accurate stress testing and supervision were not available. We included a validated 200 mFWT as a way to determine the training intensity according to the target HR zone. Research verified that the test could predict the HRmax very well, based on which it was possible to set the training zone during HR monitoring exercises [20].

The predictive HRmax model can be easily used because it is based on only one walking test and does not include any anthropometric criteria. Another advantage of setting a target HR zone for training is that this is close to the commonly used calculation based on the HR reserve. Although 200mFWT cannot replace the cardiopulmonary exercise test to measure the maximum exercise capacity, it brings a suitable alternative in evaluating people with CHD, which can calculate a target HR and set limits on the maximum intensity of physical exercise [39].

### 4.1. Impacts of Research

Our research evidence supports the effectiveness of telerehabilitation, which may impact addressing access barriers during the COVID-19 pandemic. Our findings showed that telerehabilitation interventions bring beneficial effects for health and can meet the needs of many people with CHD. At the same time, the HBCT method can optimize the CR and the number of participants, especially for those who have a low risk of complications.

The advantage of a telerehabilitation approach is cost-effectiveness. Implementing telerehabilitation can reduce healthcare costs and socio-economic costs. Telerehabilitation has been shown to be at least as cost-effective as center-based CR [36]. The data from clinical trials show a reduced inability to work and rate of rehospitalization in participants after HBCT compared to center-based CR ([36,40]). Reducing healthcare costs can serve as an important argument for the large-scale implementation of telerehabilitation, especially because the limited number of training facilities and low budgets in CR centers represent a limitation in providing rehabilitation to all suitable participants.

The research highlighted ICT possibilities and innovations in telemedicine. Our study shows the willingness of older people diagnosed with CHD to engage in new technologies. We expected that operating more technically complex devices for monitoring physical exercise and data sharing would be a barrier for older people (60+) who were likely to be less able to operate ICT than the younger population. However, the findings show that, if necessary, this age group is already sufficiently literate to use ICT in CR. In this respect, there may no longer be a cause for concern that telemedicine technologies are an obstacle for older people with CHD.

### 4.2. Limitations

One of the limitations is the fact that this was a study of one center. Due to the COVID-19 pandemic and restrictions corresponding to the minimization of social contacts, a control group was absent from the study. Although our work has shown that telerehabilitation based on a walking test is useful, it has not answered whether this training is better or worse than other methods used (e.g., usual care). This limitation may be the subject of further studies

The study’s sample of female participants was relatively small compared to male participants. Therefore, we cannot generalize our results for the entire population of people with CHD. Future studies with larger sample sizes are needed to refine our research.

Finally, one issue that remains to be discussed is the acceptable error rate in the HRmax prediction. The intensity of physical exercise based on the HR zone calculation using 200 mFWT could be a source of bias due to the standard deviation. Predictive studies reported a possible deviation of HRmax in the range of 10 to 22 bpm, which was evaluated as an acceptable error rate in the prediction. Therefore, it is a new approach in predicting HRmax and should be further confirmed in a larger number of participants with cross-validation.

## 5. Conclusions

HBCT, based on the 200 mFWT, effectively increased the cardiorespiratory fitness in people with CHD with a low to moderate cardiovascular risk. The study showed that telerehabilitation based on a walking test and telemonitoring via an HR monitor could become an alternative to CR. This was a novel approach during the COVID-19 pandemic. Wearable sensors can reliably transmit data to remote monitoring locations via ICT, bringing the universal availability of supervision closer to CR specialists. As research in this area is justified, this sheet may serve as an alternative method of providing healthcare during the COVID-19 pandemic and as a basis for further upcoming randomized controlled trials.

## Figures and Tables

**Table 1:** Baseline characteristics of the study participants.

Characteristics	Participants (*n* = 19)
Age (years)	60.4 ± 9.6
Male sex, *n* (%)	14 (74)
Weight (kg)	87.6 ± 17.9
Height (cm)	175.4 ± 9.0
**Diagnosis**	
AP, *n* (%)	2 (11)
AMI, *n* (%)	17 (90)
**Intervention**	
PCI, *n* (%)	19 (100)
LVEF (%)	58.5 ± 7.7
**Medication**	
ACEi, *n* (%)	16 (84)
Statins, *n* (%)	16 (84)
Antiplatelets, *n* (%)	19 (100)
Betablockers, *n* (%)	18 (95)
Diuretics, *n* (%)	5 (26)
Antidiabetics, *n* (%)	3 (16)
**Risk factors**	
Hypertension, *n* (%)	11 (58)
Diabetes, *n* (%)	4 (21)
Dyslipidemia, *n* (%)	11 (58)
Smoking, *n* (%)	9 (47)
Family history, *n* (%)	10 (53)
BMI (kg/m2)	28.5 ± 5.6

Values expressed as mean ± standard deviation, number of participants or *n* (%). Abbreviations: ACEi, angiotensin-converting enzyme inhibitors; AP, angina pectoris; AMI, acute myocardial infarction; BMI, body mass index; LVEF, left ventricular ejection fraction; and PCI, percutaneous coronary angioplasty.

**Table 2:** Effect of home-based cardiac telerehabilitation intervention on the 200 mFWT.

Parameters	Baseline (T1)	8-Weeks (T2)	Z-Value	*p*-Value
Time of 200 mFWT (s)	113.6 (10.4)	104.8 (7.9)	−3.361	0.001
HRmax of 200 mFWT (bpm)	106.2 (12.5)	108.3 (9.8)	−1.476	0.138
HRrest (bpm)	71.4 (10.9)	69.4 (8.8)	−1.215	0.222
RPE 6–20 (grade)	14.7 (2.0)	13.9 (1.7)	−1.817	0.068

Values expressed as mean (standard deviation). Abbreviations: 200 mFWT, 200 m fast walk test; bpm, beats per minute, HRmax, maximal heart rate recorded at the end of the 200 mFWT; HRrest, resting heart rate recorded 6 min after the 200 mFWT; and RPE, rate of perceived exhaustion on the Borg scale 6–20.

## Data Availability

The data presented in this study are available in the article or supplementary material.

## Supplementary Materials

The following are available online at https://www.mdpi.com/1660-4601/18/5/2241/s1.
