# Supplementary material for: Cardiac Rehabilitation Based on the Walking Test and Telerehabilitation Improved Cardiorespiratory Fitness in People Diagnosed with Coronary Heart Disease during the COVID-19 Pandemic
Source: Int J Environ Res Public Health. 2021 Feb 24;18(5):2241. doi: 10.3390/ijerph18052241 (PMC7956401; doi:10.3390/ijerph18052241)
Supplement: Supplementary file 1 [file ijerph-18-02241-s001.pdf]

109	97	86	94	67	63	17	15
113	109	120	120	89	83	16	13
104	96	107	116	80	86	15	15
117	112	107	112	78	79	15	13
117	105	95	102	58	63	14	13
124	110	107	102	70	63	15	16
129	112	121	116	92	76	17	15
115	101	133	123	83	78	18	15
120	103	89	92	78	75	10	9
120	105	104	100	63	67	15	15
100	94	110	106	70	66	13	15
122	118	97	104	69	68	12	13
126	117	99	104	59	63	12	13
111	107	120	126	64	59	16	15
95	99	100	107	55	57	15	15
96	92	104	108	67	65	15	13
seconds		HRmax		HRrest		RPE	
